# Health Tract No. 316

**Published:** 1868-01

**Authors:** 


### THE WORLD'S WORTHIES.

Not the Cæsars of the ages, any more than their Attilas ; not the Bonapartes, nor the Swedish Charles, nor the Dutch Frederick, are they; their paths to glory and renown were laid through seas of blood and rapine; fire and sword; she is worthier, who at eleven, took charge of her father's motherless children; and without an education; without experience; without counsel beyond the four walls of her own homely hut, became to these little ones a care-taker and a guide, for long successive years; when they were out of the way, she married; and by industrious economies, and faithful and tireless and willing co-operation in her husband's plans, lands were bought and paid for, and by her counsels were retained in the family, against the urgent advice of older and wiser and more experienced persons; she was not learned; she could scarcely write her name; her language was homely, and her grammar bad; in fact, so "illiterate" was she, that some who were nearest to her, did not care, many times, to compromise themselves, by introduction to their friends, of high social position ; but the price of that land has reared an institution for preparing young girls for domestic life, second to none of its kind on the habitable globe, so liberally is it founded and so complete in its appointments—"The Vassar Female College" of Pokeepsie, which ought to have been called "The Catherine Vassar College."

Fifty years ago, a poor boy straitened for means to procure a few volumes to read, formed the idea of founding an institution which should provide books and papers and schooling, without charge for all who desired it ; later on, he presented such an establishment to the city of New-York, at an expense to himself of over six hundred thousand dollars, and the name of

PETER COOPER

will go down to the coming ages, with imperishable honor.

More than half a century since, a poor, one-eyed German youth stepped ashore at Philadelphia from a ship in the Delaware, an uneducated, unnoticed and unknown stranger, almost penniless; later on, he gave millions to the city of his adoption, and by the beneficence of

STEPHEN GIRARD, four hundred houseless, homeless, orphan babes, are picked out of the gutters of the streets, and are clothed and fed and sheltered and educated and put out to a useful trade; and then another four hundred are taken thus; and so on will it be, while our nation stands !

Forty years since, a Scotch-Irish youth, with a thousand dollars in his pocket, landed at Castle Garden; finding nothing better, he collected some children together and taught them their A, B, C; next he is found in a ten by twelve store, selling ribbons and laces, and the like, by day and taking them to his customer's dwellings by night; it was noticed that his goods were always equal to what he represented them to be, and people began to put confidence in him ; he rose; his business flourished; and within a year, he laid aside a million of dollars, that poor hard working girls and women might have cheap board and tidy, healthful apartments, without being demoralized by a sense of charity ; another five millions he has appropriated for cozy dwellings at a very cheap rate, for the struggling worthy poor and their liltle families. Thus has done A. T. STEWART. Such as these, reader, are the true worthies of society, for Divinity has said "By their fruits ye shall know them;" "inasmuch as ye did it unto one of the least of these my brethren, ye have done it unto me," "come up higher." It is not the pronunciation of a "Shiboleth," which entitles a man to a "mansion in the skies;" it is the great broad fact of a life consecrated to that "charity" "which covereth a multitude of sins," and without which, all other observances are as "a sounding brass or a tinkling cymbal."

